# Inhaled nitric oxide as adjunctive therapy for severe malaria: a randomized controlled trial

**DOI:** 10.1186/s12936-015-0946-2

**Published:** 2015-10-29

**Authors:** Michael T. Hawkes, Andrea L. Conroy, Robert O. Opoka, Laura Hermann, Kevin E. Thorpe, Chloe McDonald, Hani Kim, Sarah Higgins, Sophie Namasopo, Chandy John, Chris Miller, W. Conrad Liles, Kevin C. Kain

**Affiliations:** 3-588D Edmonton Clinic Health Academy, University of Alberta, 11405 87 Ave NW, Edmonton, AB T6G 1C9 Canada; Sandra Rotman Centre for Global Health, MaRS Centre, University of Toronto, 101 College St TMDT 10-360A, Toronto, ON M5G 1L7 Canada; Global Health Uganda, Upper Paediatrics Office, Mulago Hospital, PO Box 33842, Plot 138, Upper Mawanda Road, Kawempe, Kampala Uganda; Applied Health Research Centre, St Michael’s Hospital, 250 Yonge St, 6th Floor, Toronto, ON M5T 3M7 Canada; Johns Hopkins School of Public Health, International Vaccine Access Center, 615 N Wolfe St, Baltimore, MD 21205 USA; Jinja Regional Referral Hospital, Plot 7, Nalufenya Road, Jinja, Uganda; Department of Pediatrics, Indiana University, 702 Barnhill Dr, Room 5900, Indianapolis, IN 46202 USA; Division of Infectious Disease, University of British Columbia, Rm D433, HP East, Vancouver Hospital, 2733 Heather Street, Vancouver, BC V5Z-3J5 Canada; Department of Medicine, University of Washington, 1959 NE Pacific Street, HSB RR-511, Box 356420, Seattle, WA 98195-6420 USA

**Keywords:** Nitric oxide, Endothelium, Severe malaria, Child, Randomized controlled trial

## Abstract

**Background:**

Severe malaria remains a major cause of childhood mortality globally. Decreased endothelial nitric oxide is associated with severe and fatal malaria. The hypothesis was that adjunctive inhaled nitric oxide (iNO) would improve outcomes in African children with severe malaria.

**Methods:**

A randomized, blinded, placebo-controlled trial of iNO at 80 ppm by non-rebreather mask *versus* room air placebo as adjunctive treatment to artesunate in children with severe malaria was conducted. The primary outcome was the longitudinal course of angiopoietin-2 (Ang-2), an endothelial biomarker of malaria severity and clinical outcome.

**Results:**

One hundred and eighty children were enrolled; 88 were assigned to iNO and 92 to placebo (all received IV artesunate). Ang-2 levels measured over the first 72 h of hospitalization were not significantly different between groups. The mortality at 48 h was similar between groups [6/87 (6.9 %) in the iNO group vs 8/92 (8.7 %) in the placebo group; OR 0.78, 95 % CI 0.26–2.3; p = 0.65]. Clinical recovery times and parasite clearance kinetics were similar (p > 0.05). Methaemoglobinaemia >7 % occurred in 25 % of patients receiving iNO and resolved without sequelae. The incidence of neurologic deficits (<14 days), acute kidney injury, hypoglycaemia, anaemia, and haemoglobinuria was similar between groups (p > 0.05).

**Conclusions:**

iNO at 80 ppm administered by non-rebreather mask was safe but did not affect circulating levels of Ang-2. Alternative methods of enhancing endothelial NO bioavailability may be necessary to achieve a biological effect and improve clinical outcome.

Trial Registration: ClinicalTrials.gov NCT01255215

**Electronic supplementary material:**

The online version of this article (doi:10.1186/s12936-015-0946-2) contains supplementary material, which is available to authorized users.

## Background

Severe malaria due to *Plasmodium falciparum* claims 0.6–1.2 million lives annually, 86 % of whom are children in sub-Saharan Africa [1, 2]. Despite the use of highly effective anti-malarial medications, 10–30 % patients with severe malaria will die [3], highlighting the need for new adjunctive therapy. Nitric oxide (NO), with its modulating effects on host endothelial activation, is a promising agent based on pre-clinical findings and an established safety profile in clinical practice [4, 5].

NO is a gaseous, lipid-soluble, free radical, endogenously produced from l-arginine and molecular oxygen by members of the nitric oxide synthase family [6]. NO regulates a broad range of physiologic and pathologic processes, including vasodilation, platelet aggregation, apoptosis, inflammation, chemotaxis, neurotransmission, antimicrobial defence, and endothelial activation [6].

The vascular endothelium plays a central role in the pathogenesis of severe malaria. Parasitized erythrocytes (PEs) adhere to the endothelial cells via constitutive and cytokine-inducible receptors. NO decreases endothelial cell adhesion molecule expression, and has been shown to reduce the adherence of PEs to endothelial cells [7]. Upon activation, endothelial cells release angiopoietin-2 (Ang-2) from intracellular Weibel-Palade bodies (WPB) storage granules [8]. Ang-2 functions as an autocrine regulator by sensitizing the endothelium to the effects of tumour necrosis factor, resulting in increased adhesion receptor expression [9]. Ang-2 is associated with malarial retinopathy and is an independent and quantitative marker of disease severity and clinical outcome in malaria [10–12]. Ang-2 has also been used to follow disease progression and recovery in previous studies of malaria [13]. NO inhibits the exocytosis of WPB contents through S-nitrosylation of critical regulatory proteins [14].

Reduced bioavailability of NO contributes to the pathogenesis of severe malaria. African children with severe malaria have impaired production of NO [15] and low plasma arginine levels [16], the substrate for NO synthesis. Treatment of Indonesian adults with severe malaria with intravenous l-arginine increased levels of exhaled NO, and reversed malaria-associated endothelial dysfunction [17]. Pathways of endothelial activation and dysfunction are reflected in experimental models of severe malaria, where inhaled NO enhances endothelial integrity, reduces parasite accumulation in the brain vasculature, and improves survival [5, 18].

Administration of exogenous NO at 5–80 ppm is approved for use by the US FDA for neonates with hypoxic respiratory failure [19].Inhaled nitric oxide (iNO) has been safely used in clinical practice and in large clinical trials involving critically ill neonates [19], as well as in children and adults with refractory hypoxia [20]. Adverse effects attributable to iNO in these studies are rare and include methaemoglobinaemia, renal insufficiency [21] and hypotension.

To test the hypothesis that iNO would improve outcomes in children with severe malaria, a randomized, blinded, controlled trial was conducted. The primary objective was to determine if iNO at 80 ppm, relative to placebo room air, would improve endothelial function as determined by an accelerated rate of decline of Ang-2 in peripheral blood among African children with severe malaria receiving artesunate.

## Methods

### Trial design

This was a prospective, parallel arm, randomized, placebo-controlled, blinded trial of iNO versus placebo (1:1 ratio), among children with severe malaria, all of whom were treated with artesunate. The trial protocol has been described in detail previously [22].

### Ethics, consent and permissions

The study was reviewed and approved by the Makerere University School of Medicine Research Ethics Committee (REC Protocol # 2010-107), the Uganda National Council on Science and Technology (Ref: HS 857), the National Drug Authority of Uganda (Ref: 297/ESR/NDA/DID-01/2011), and the University Health Network Research Ethics Committee, Toronto, Canada (UHN REB Number 10-0607-B). A data and safety monitoring board (DSMB) was convened and met periodically to review trial quality and adverse events. An interim analysis at the trial midpoint was conducted to review trial quality and safety, at which time the DSMB recommended that the trial proceed without modifications. The trial is registered (ClinicalTrials.gov Identifier: NCT01255215).

### Setting and participants

The trial was conducted at a single centre, the Jinja Regional Referral Hospital, in Uganda. Malaria transmission is moderate and seasonal in Jinja and the surrounding Busoga catchment area [23]. The hospital operates under severe resource constraints, and over 30 % of all admissions are due to malaria.

Children (age 1–10 years) were included if they had a positive rapid diagnostic test for both *P. falciparum* histidine rich protein 2 (HRP2) and lactate dehydrogenase (pLDH)(First Response Malaria Ag. (pLDH/HRP2) Combo Rapid Diagnostic Test, Premier Medical Corporation Limited, India) [24], as well as selected criteria for severe malaria: repeated seizures (two or more generalized seizures in 24 h), prostration, impaired consciousness (Blantyre Coma Score <5), respiratory distress (age-related tachypnea with sustained nasal flaring, deep breathing or sub-costal retractions). Patients were not included if they had methaemoglobin (metHb) >2 % at baseline, known chronic illness (renal, cardiac or hepatic disease, diabetes, epilepsy, cerebral palsy, or AIDS), severe malnutrition (weight-for length or height below −3 standard deviations based on WHO reference charts, or symmetrical oedema involving at least the feet). Modifications to the exclusion criteria were made with regulatory committee approval after experience with the first 20 enrolled participants. The following exclusion criteria were added: haemoglobinopathy, clinical suspicion of acute bacterial meningitis, unlikely to tolerate mask for study gas delivery, and prior quinine in the emergency department. Trial nurses or clinicians from the emergency department screened patients for eligibility using a uniform checklist and clinicians made final decisions about inclusion in the study.

### Randomization and blinding

In order to blind clinicians, nurses, parents, and participants to treatment while titrating and monitoring concentrations of iNO and dose-related levels of metHb and NO_2_, a dedicated unblinded team was used, the members of which were not involved in clinical care decisions or outcome assessments.

Eligible patients were randomly assigned to treatment with either iNO or room air placebo (both arms received intravenous artesunate). Simple randomization was employed, using a computer-generated list created by unblinded team leader (AC) prior to trial commencement. Treatment assignment was recorded on paper and kept in sequentially numbered, sealed, opaque envelopes in a locked cabinet accessible only to the unblinded study team. After patient stabilization and informed consent, the next envelope was drawn by an unblinded investigator.

iNO was indistinguishable from room air in colour and delivery apparatus (mask, tubing, a stream of vehicle air). An unblinded team member initiated the study gas while treating nurses and clinicians were out of the room. Flowmeters and monitoring devices were in locked opaque boxes accessible only to the unblinded study team. MetHb measurements were performed using non-invasive pulse CO-oximetry (Masimo Rad-57™, Masimo Corporation, Irvine, CA, USA) by unblinded study team members. All laboratory assays and statistical analyses were performed blinded to treatment allocation.

### Procedures

iNO was delivered continuously at a target concentration of 80 ppm by non-rebreather mask for up to 72 h. An air compressor was used to deliver continuous flow of vehicle air, and NO from compressed cylinders was titrated into the air stream to a concentration of 80 ppm, measured continuously at the bedside using a NO-NO_2_ analyser (Pulmonox Sensor; Pulmonox Research and Development Corporation, Tofield, Alberta, Canada). Methaemoglobinaemia and inspired NO_2_ were monitored at least every 4 h. The concentration of iNO administered was adjusted downward if the metHb level in peripheral blood rose above 7 %, and was temporarily discontinued for metHb >10 %. Participants in the control group received room air by non-rebreather mask. Both groups received intravenous artesunate, the recommended first-line treatment for severe malaria, at recommended dose and frequency [25]. Follow-on oral therapy was with artemether-lumefantrine tablets or suspension for 3 days.

Bloodwork for clinical and study purposes was drawn at admission and daily during the first 72 h of hospital admission. Admission venous blood samples were analysed at the bedside for haematocrit, creatinine, lactate, and glucose [26] and at a central laboratory for parasite density, as previously described [24]. Lumbar puncture was performed at the clinician’s discretion and was analysed for cell count and differential, total protein, Gram stain and bacterial culture.

### Study outcomes

The analysis was undertaken according to a pre-specified analytical plan [22]. There were no changes to any trial outcomes after the trial commenced. The primary endpoint was the longitudinal serum Ang-2 concentration over the first 72 h of hospital admission. Ang-2 was measured from serum samples using commercially available enzyme-linked immunosorbent assay (ELISA) kits (DuoSets, R&D Systems, Minneapolis, MN, USA).

Secondary trial outcomes included: mortality, recovery times, parasite clearance kinetics, and safety. Adverse events were monitored daily using paediatric toxicity tables modified from the US National Institute of Allergy and Infectious Diseases [27].

### Statistical analysis

Inclusion of 180 children with severe malaria was needed to show, with 80 % power and 95 % confidence, a 50 % difference in the rate of change of Ang-2. This calculation was supported by a simulation study under various assumptions of variance and treatment effect [22].

The primary outcome, longitudinal course of Ang-2, was compared between study arms using linear mixed-effects (LME) models. All available data was used for the primary analysis. Because of (non-random) missing longitudinal data due to death, withdrawal and lost samples, sensitivity analyses were performed with different methods of adjusting for missing data (‘intention-to-treat’ analysis), as outlined in Additional file 1. Model fit was assessed by visual inspection of residuals.

For secondary binary outcomes, Chi squared or Fisher exact test were used, as appropriate. Time to event outcomes were compared with the log-rank test, and hazard ratios (HRs) together with 95 % CIs were estimated by a Cox proportional hazard model.

Statistical analyses were done with SPSS (version 16.0) and R (version 3.0.1).

### Role of the funding source

The sponsor of the study had no role in study design, data collection, data analysis, data interpretation, or writing of the report.

## Results

**Fig. 1:**
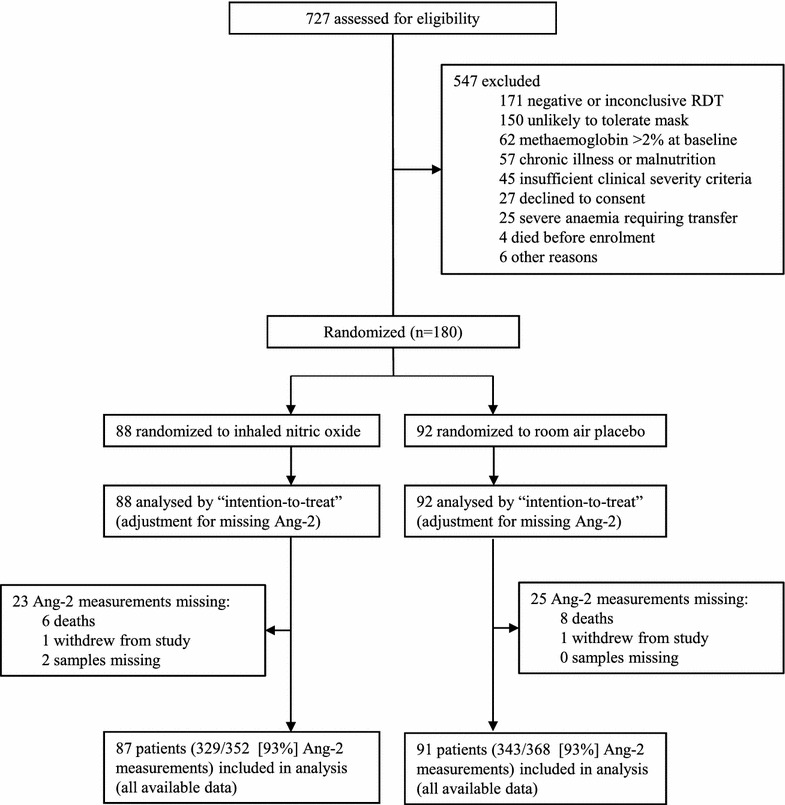
Trial profile. *RDT* rapid diagnostic test, *Ang-2* angiopoietin-2, *LME* linear mixed effects

Figure 1 shows the trial profile. Recruitment occurred between 12 July, 2011 and 14 June, 2013, with last follow-up visit on 28 June, 2013. The trial ended when the pre-specified sample size was reached.

No significant differences in baseline characteristics between the two treatment groups were observed (Table 1). Ten patients were positive for both HRP2 and pLDH bands on screening rapid diagnostic test but negative on microscopy of the admission sample. One additional patient had a microscopist diagnosis of *Plasmodium ovale*. All these samples tested positive for *P. falciparum* by PCR. These cases were equally distributed between groups (5/88 (5.7 %) iNO and 6/92 (6.5 %) placebo, p = 1.0). No alternative diagnosis was apparent (blood culture negative in all cases) and two patients with negative microscopy died.

**Table 1:** Baseline characteristics of the two treatment groups

	iNO (N = 88)	Placebo (N = 92)
Female sex	35 (40 %)	43 (47 %)
Age (years), median (IQR)	2.0 (1.0–3.0)	2.0 (1.0–3.0)
Fever before enrolment (days), median (IQR)	3 (2–4)	3 (2–4)
Coma before enrolment (hours), median (IQR)	8.0 (3.0–11)	5.0 (2.0–15)
Pretreatment with anti-malarials		
None	36 (41 %)	42 (46 %)
Ineffective^a^	5 (6 %)	1 (1 %)
Effective^a^	47 (53 %)	49 (53 %)
Complications on admission		
Coma	54 (61 %)	52 (57 %)
Convulsions	69 (78 %)	75 (82 %)
Jaundice	13 (15 %)	15 (16 %)
Severe anaemia (haemoglobin <50 g/L)	44 (50 %)	51 (55 %)
Shock	9 (10 %)	12 (13 %)
Hypotensive shock	1 (1 %)	1 (1 %)
Severe acidosis (BE <−8 mmol/L)	36 (50 %)	33 (44 %)
Hypoglycaemia (<3 mmol/L)	4 (5 %)	7 (8 %)
Respiratory distress^b^	50 (57 %)	46 (50 %)
Prostration	78 (89 %)	86 (93 %)
Haemoglobinuria	18 (21 %)	16 (17 %)
Hyperparasitaemia (>10 %)	10 (11 %)	7 (8 %)
Clinical examination		
Weight (kg), mean (SD)	11.5 (3.7)	11.7 (3.6)
Temperature (°C), mean (SD)	38.0 (1.2)	37.9 (1.1)
Blood pressure (mm Hg)		
Systolic, mean (SD)	108 (20)	113 (20)
Diastolic, mean (SD)	58 (14)	60 (13)
Blantyre coma score, median (IQR)	2 (2–3)	2 (2–3)
Co-morbidity		
HIV	4 (5 %)	1 (1 %)
Suspected pneumonia^c^	8 (9 %)	12 (13 %)
Clinical sepsis^d^	27 (31 %)	28 (30 %)
Suspected meningitis^e^	4 (5 %)	1 (1 %)
Laboratory assessments		
Parasitaemia (parasites per μL), geometric mean (range)^f^	15,700 (0–696,000)	19,300 (0–316,000)
Sodium (mmol/L), mean (SD)	139 (5.0)	137 (4.6)
Potassium (mmol/L), mean (SD)	4.2 (0.6)	4.1 (0.6)
Chloride (mmol/L), mean (SD)	110 (6)	107 (5)
Creatinine (umol/L), mean (SD)	44 (33)	36 (27)
Haemoglobin (g/L), mean (SD)	61 (24)	62 (27)
pH, mean (SD)	7.37 (0.12)	7.38 (0.11)
PaCO_2_ (mm Hg), mean (SD)	28.0 (10.2)	28.1 (7.5)
HCO_3_ (mmol/L), mean (SD)	10.0 (11.6)	10.7 (12.0)
Plasma BE (mmol/L), mean (SD)	−2.8 (12.7)	−2.5 (12.2)

Data are number (%), unless otherwise indicated

*SD* standard deviation, *IQR* interquartile range, *BE* base excess, *PaCO*_*2*_ partial pressure of carbon dioxide, *HCO*_*3*_ bicarbonate

^a^Classification of anti-malarial effectiveness followed AQUAMAT [25]. The following were considered effective: quinine injection, artemether injection, artesunate/artemether tabs, artemether-lumefantrine, artesunate suppository, artesunate-amodiaquine, artemether-amodiaquine, artemether-quinine, dihydroartemisinin (DHA), DHA-amodiaquine, and SP-artemether-lumefantrine. The following were considered intermediate or ineffective: sulfadoxine-pyrimethamine (SP), SP-amodiaquine, chloroquine, amodiaquine, and pyrimethamine-sulphamethopirazine

^b^Respiratory distress was defined as nasal alar flaring, costal indrawing, or deep breathing

^c^Pneumonia was diagnosed on clinical grounds (chest radiographs were not performed routinely). Fast breathing and fever were signs used to make a diagnosis of pneumonia

^d^Blood cultures were positive in 2/88 (2.3 %) iNO group and 8/91 (8·8 %) placebo: 6 coagulase-negative staphylococcus (likely contaminants); 2 *Staphylococcus aureus*; and 2 coliform Gram-negative organism. Of note, antibiotics had been given prior to venipuncture for blood culture in almost all cases

^e^Lumbar puncture was performed in 23/88 (26 %) iNO group and 28/92 (30 %) placebo. CSF culture was negative in all patients but one, which grew mixed Gram positive and Gram-negative organisms (likely contamination). The CSF leukocyte count was <5 cells/mm^3^ in all but one patient, who had 35 cells (predominantly lymphocytes) and negative blood culture

^f^In 10 cases, no parasites were seen on the admission blood smear, despite a positive screening RDT result. In one other case, the species was diagnosed as *P. ovale*. In all of these cases, PCR performed on the red blood cell pellet was positive for *P. falciparum* [24]

**Fig. 2:**
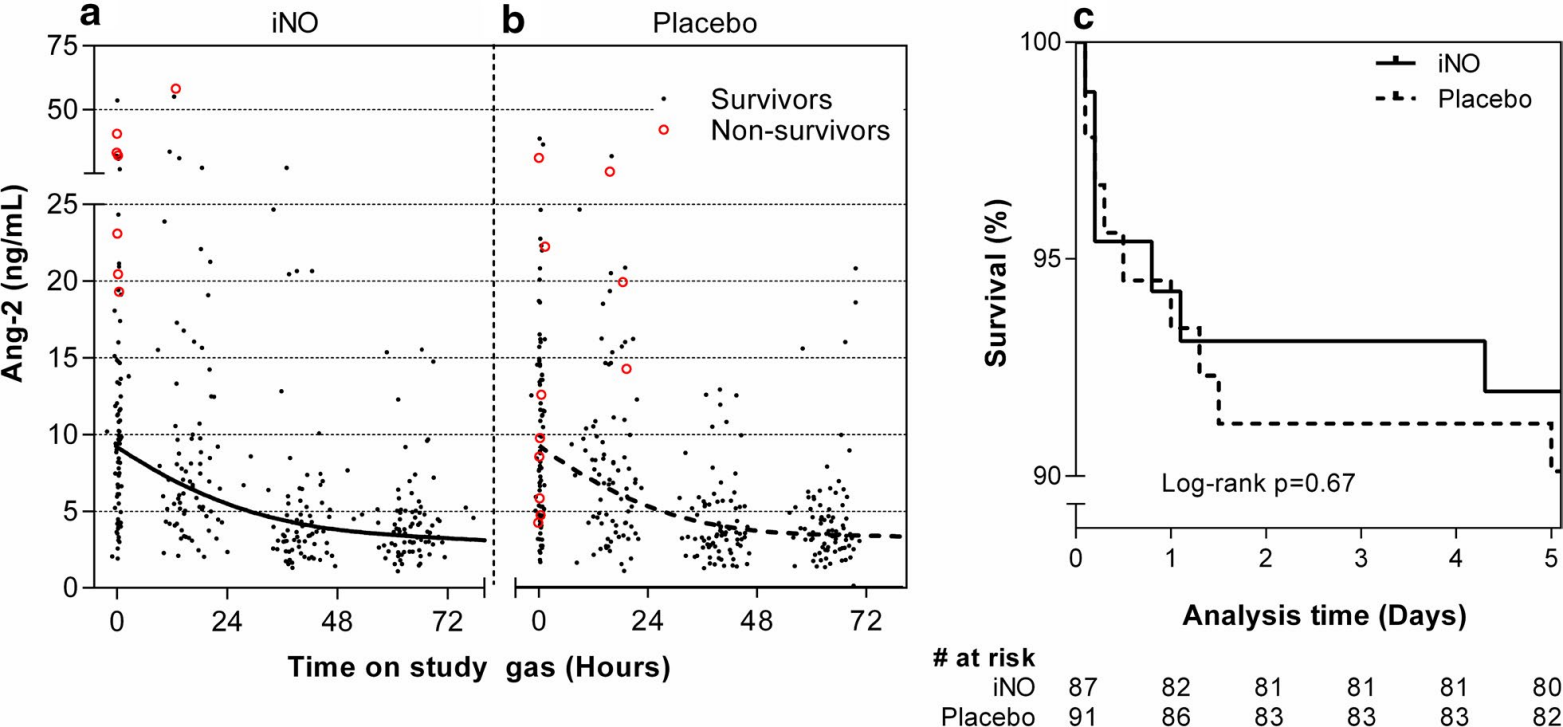
Primary biochemical trial endpoint and mortality. Longitudinal Ang-2 concentrations in treatment (iNO) and control (room air) groups did not differ significantly (p = 0.72, **a** and **b**). *Solid black dots* (indicating survivors), *red circles* (non-survivors), and best-fit curve from a LME model are shown. Survival curves were not significantly different between groups (p = 0.67, **c**)

The median (IQR) linear rate of change of Ang-2 over the first 72 h of hospitalization was −2.1 (−3.1 to −1.2) ng/mL/day in patients receiving iNO vs −1.9 (−3.6 to −0.57) ng/mL/day in patients receiving placebo (p = 0.68). A LME model did not show a statistically significant effect of iNO on the rate of change of Ang-2 over time (p = 0.72). Figure 2a, b shows the Ang-2 levels over time for patients in the iNO and placebo groups, together with best fit curve from the LME model.

Mortality outcomes (Table 2) and survival curves (Fig. 2c) show that most deaths occurred within 48 h of admission and occurred at similar frequency in both groups. Incidence of complications during hospitalization (development of coma, deterioration of coma score, new or persistent seizures, hypoglycaemia, development of severe anaemia, and haemoglobinuria) was also similar between groups (Table 2). Ang-2 levels were higher at admission among patients who subsequently died compared to survivors (median (IQR) 20 (8.8–29) ng/mL among fatal cases vs 8.9 (4.9–14) ng/mL among survivors; p = 0.0068). Co-treatments, administered by physicians blinded to randomization arm, were similar between groups, with the exception of anticonvulsants (Table 2). In particular, the use of antibiotics was not statistically significantly different between groups (82/87 (94 %) iNO and 80/91 (88 %) placebo, p = 0.19).

**Table 2:** Mortality, complications and co-treatments according to treatment group

	iNO (n/N, %)	Placebo (n/N, %)	OR (95 % CI)	p value
Mortality				
Mortality, 48 h	6/87 (6.9 %)	8/92 (8.7 %)	0.78 (0.26–2.3)	0.65
Mortality, in hospital	7/87 (8.0 %)	8/92 (8.7 %)	0.92 (0.32–2.7)	0.88
Mortality, 14 days	7/87 (8.0 %)	9/91 (9.9 %)	0.80 (0.28–2.2)	0.67
Death or sequelae at 14 days	12/87 (13 %)	16/91 (14 %)	0.75 (0.33–1.7)	0.75
Mortality in strictly defined severe malaria^a^	7/81 (8.6 %)	9/88 (10 %)	0.83 (0.29–2.3)	0.73
Mortality in strictly defined cerebral malaria^b^	6/53 (11 %)	8/51 (16 %)	0.69 (0.22–2.1)	0.51
Mortality in HIV	1/4 (25 %)	0/1 (0 %)	–	1.0
Complications				
Development of coma^c^	2/26 (7·7 %)	0/34 (0 %)	–	0.18
Deterioration of coma score	8/86 (9·3 %)	5/91 (5·5 %)	1·8 (0·55–5·6)	0.33
Convulsions developing or persisting >6 h after admission	19/87 (22 %)	13/91 (14 %)	1.7 (0·77–3·6)	0.19
Hypoglycaemia (<3 mmol/L)	10/88 (10 %)	17/91 (19 %)	0.56 (0·24–1·3)	0.12
Severe anaemia (<50 g/L) after admission^c^	11/35 (31 %)	5/34 (15 %)	2·7 (0.81–8.7)	0.10
Haemoglobinuria^c^	0/68 (0 %)	3/76 (3.9 %)	0 (0–3.6)	0.25
Co-treatments				
Blood transfusion	65/87 (75 %)	67/91 (74 %)	1.1 (0.54–2.1)	1.0
Intravenous fluids	80/88 (91 %)	82/91 (90 %)	1.1 (0.40–3.0)	1.0
Antibiotics (any)	82/87 (94 %)	80/91 (88 %)	2.3 (0.75–6.8)	0.19
Ceftriaxone	81/87 (93 %)	76/91 (84 %)	2.7 (0.98–7.2)	0.06
Anticonvulsant (any)	51/87 (59 %)	36/91 (40 %)	2.2 (1.2–3.9)	0.016
Diazepam	40/87 (46 %)	27/91 (30 %)	2.0 (1.1–3.7)	0.030
Phenobarbitone	29/87 (33 %)	23/91 (25 %)	1.5 (0.77–2.8)	0.25
Antipyretic (any)	64/87 (74 %)	71/91 (78 %)	0.78 (0.39–1.6)	0.60

*OR* odds ratio

^a^Strictly defined severe malaria as in AQUAMAT [25]. At least one of the following criteria was required: plasma base excess <–3.3 mmol/L; Blantyre coma scale <3/5; haemoglobin <50 g/L and parasitaemia >100,000 parasites/μL; compensated shock (capillary refill ≥3 s but no hypotension); decompensated shock (systolic blood pressure <70 mmHg and cool peripheries); asexual parasitaemia >10 %; visible jaundice and >100,000 parasites/μL; plasma glucose <3 mmol/L; respiratory distress (defined as costal indrawing, use of accessory muscles, nasal alar flaring, or deep breathing)

^b^Strictly defined cerebral malaria required Blantyre coma scale <3, exclusion of hypoglycaemia and post-ictal state as the cause of coma

^c^Development of coma, anaemia and haemoglobinuria was assessed only in patients without these disorders on admission

Among survivors, the recovery times (time to eat, sit unsupported, localize pain, fever resolution, recovery of consciousness, and discharge) were similar between groups (Table 3). Parasite clearance kinetics with artesunate were unaffected by iNO (Table 3). No parasite recrudescence or re-infection was detected at day 14 follow-up in either group. At the time of discharge, 5/88 (5.7 %) iNO recipients and 8/92 (8.7 %) placebo recipients had neurologic deficits, including inability to sit, spastic or flaccid paresis of one or more limbs, seizures, unilateral weakness, vision loss, gaze palsy, and poor head control.

**Table 3:** Recovery times in surviving patients according to treatment group

	iNO (N = 80) (median, IQR)	Placebo (N = 83) (median, IQR)	HR (95 % CI)	p value
Time to discharge (days)	4.0 (3.0–4.0)	3.0 (3.0–4.0)	1.1 (0.83–1.6)	0.40
Time to eat (h)	17 (7–37)	10 (6–23)	1.3 (0.92–1.7)	0.15
Time to sit unsupported (h)	41 (20–75)	28 (13–60)	1.3 (0.98–1.8)	0.063
Time to localize pain (h)	5 (0–11)	2 (0–9.6)	1.2 (0.84–1.6)	0.38
Time to fever resolution (h)	6 (0–31)	6 (0–23)	1.1 (0.78–1.4)	0.73
Time to recovery of consciousness (h)	14 (6–36)	9 (4–19)	1.2 (0.90–1.7)	0.19
Time to 50 % parasite clearance (h)	12 (9–16)	12 (9–18)	0.87 (0.63–1.2)	0.37
Time to 90 % parasite clearance (h)	19 (12–37)	20 (14–37)	0.85 (0.62–1.2)	0.31
Time to parasite clearance (h)	44 (35–63)	44 (37–63)	0.97 (0.71–1.3)	0.83

*IQR* inter-quartile range, *HR* hazard ratio

Adverse events were systematically recorded using standardized tables on a daily basis in all patients (Table 4). The study gas was temporarily or permanently discontinued in 19/88 (22 %) iNO patients *vs* 12/92 (13 %) placebo (p = 0.13) for reasons shown in Table 5. In 5/88 (5.7 %) patients receiving iNO, study gas was temporarily discontinued because of elevated metHb levels. In all cases, iNO was resumed after metHb levels declined and iNO therapy did not need to be permanently discontinued in any patient due to recurrent or refractory methaemoglobinaemia. The study gas did not need to be titrated downward or discontinued in any patient for elevated inhalational levels of NO_2_.

**Table 4:** Adverse events according to treatment group

Adverse event	iNO (n/N, %)	Placebo (n/N, %)	p value
Hyperglycaemia (> 6.8 mmol/L)	34/88 (39)	29/92 (32)	0.32
Hypoglycaemia (< 3 mmol/L)	5/88 (5.7)	2/92 (2.2)	0.27
Elevated creatinine	30/88 (34)	24/92 (26)	0.26
Grade 1^a^	8/88 (9.1)	8/92 (8.8)	1.0
Grade 2^a^	11/88 (13)	10/92 (11)	0.82
Grade 3^a^	3/88 (3.4)	3/92 (3.3)	1.0
Grade 4^a^	8/88 (9.1)	3/92 (3.3)	0.13
Acute kidney injury^b^	7/88 (8.0)	3/92 (3.3)	0.21
Hypotension	0	0	–
Anaemia	9/88 (10)	6/92 (6.6)	0.38
IV Site oedema or induration	17/88 (19)	10/92 (11)	0.12
Peri-orbital oedema	17/88 (19)	4/92 (4.4)	0.002
Vomiting	4/88 (4.5)	4/92 (4.4)	1.0
Conjunctivitis	1/88 (1.1)	1/92 (1.1)	1.0
Diarrhoea	5/88 (5.7)	4/92 (4.4)	0.74
Fever	4/88 (4.5)	5/92 (5.4)	1.0
Cough	5/88 (5.7)	5/92 (5.4)	0.94
Stridor	0	2/92 (2.2)	0.50
Cellulitis	4/88 (4.5)	1/92 (1.1)	0.20
Headache	0	1/92 (1.1)	1.0
Jaundice	1/88 (1.1)	3/92 (3.3)	0.62
Other	14/88 (16)	7/92 (7.7)	0.11
Withdrawal of study gas	19/88 (22)	13/92 (14)	0.13

^a^Elevated creatinine was defined as follows: For children age 1 to <2 years old, grade 1: 0.6–0.89 × upper limit of normal (ULN); grade 2: 0.9–1.19 × ULN; grade 3: 1.2–1.5 × ULN, grade 4: >1.5 × ULN. For children 2–10 years old, grade 1: 0.7–1.0 × ULN; grade 2: 1.1–1.6 × ULN; grade 3: 1.7–2.0 × ULN, grade 4: >2.0 × ULN [27]

^b^Acute kidney injury was defined as follows: Serum creatinine >1.5 × ULN (children age 1–2) or >2.0 × ULN (age 2–10) AND an abrupt (within 48 h) reduction in kidney function: (1) an absolute increase in serum creatinine of ≥26.4 μmol/l; or (2) a percentage increase in serum creatinine of ≥50 % [32]

**Table 5:** Study gas discontinuation according to treatment group

Adverse event	iNO (n/N, %)	Placebo (n/N, %)	p value
Temporary discontinuation	7/88 (8.0)	3/92 (3.3)	0.21
Methaemoglobinaemia	5/88 (5.7)	0	0.026
Elevated inspired NO_2_ concentration	0	0	–
Persistent hypoxaemia	0	0	–
Evolving respiratory distress	0	0	–
Unexplained tachycardia	1/88 (1.1)	0	0.49
Unexplained hypotension	0	0	–
Subject or guardian’s discretion	0	0	–
Investigator’s discretion	1/88 (1.1)	3/92 (3.3)	0.62
Permanent discontinuation	12/88 (14)	10/92 (11)	0.57
Refractory methaemoglobinaemia	0	0	–
Haemoptysis	0	0	–
Acute kidney injury	7/88 (8.0)	3/92 (3.3)	0.21
Adverse event that repeats upon re-challenge	0	0	–
Subject or guardian’s discretion^a^	4/88 (4.5)	4/92 (4.3)	1.0
Investigator’s discretion^a^	1/88 (1.1)	2/92 (2.2)	1.0
Death (48 h)	6/88 (6.8)	8/92 (8.8)	0.62
Death (Day 14)	7/88 (8.0)	9/92 (9.9)	0.65

^a^Some children who regained consciousness before 72 h had elapsed on study gas did not tolerate wearing a non-rebreather mask. The study gas was discontinued early at the investigator’s or the child/parent’s discretion in these cases

Evaluation of the quality of blinding and indices of the quality of clinical care provided in the trial are provided in Additional file 1.

## Discussion

iNO at 80 ppm (iNO_80_) by non-rebreather mask was safe but did not accelerate the decline in circulating Ang-2 levels during the first 72 h of hospitalization in this study of African children with severe malaria. No differences in clinical outcomes (e.g., mortality, recovery times) were observed, likely because a measurable biological effect on the endothelium was not achieved with this dose and route of administration of NO. Of note, parasite clearance under artesunate treatment was not affected by the addition of iNO.

Among survivors of severe malaria in this study, the rate of change of Ang-2 over the first 72 h of hospitalization was −2.2 ng/mL/day (iNO group) and −1.9 ng/mL/day (placebo group), very similar to that observed in a study involving Indonesian adults, at −2.7 ng/mL/day [13]. Among four patients who died but had repeated measurements of Ang-2, the level increased on average by +15 ng/mL/day, compared to +9.5 ng/mL/day in the study from Indonesia [13]. Moreover, elevated Ang-2 levels at admission were strongly predictive of mortality in this study and in several previous reports from several populations [12, 26, 28, 29]. These findings lend further validity to Ang-2 as a clinically informative biomarker for prognosis in children with severe malaria that can be used to follow the course of disease progression and as a surrogate endpoint for severe malaria studies.

In-hospital mortality was similar in the present trial to the largest published clinical trial of paediatric severe malaria to date, AQUAMAT [25]. In the placebo arm of the present trial (patients receiving artesunate alone) the mortality was 8.7 %, compared to 8.5 % in the artesunate arm of AQUAMAT. Mortality was similar despite evidence that patients in the present trial had more severe disease at presentation: 57 vs 32 % coma; 82 vs 30 % convulsions; 55 vs 30 % severe anaemia; 50 vs 16 % respiratory distress; 93 vs 62 % severe prostration, in the placebo arm of the present trial vs artesunate arm of AQUAMAT, respectively [25]. The clinical care in the present trial (see Additional file 1) was superior to that provided at Uganda’s national referral hospital by several indices: (1) 77 vs 23 % were seen by a clinician within one hour of presentation; (2) 68 vs 12 % received the first dose of anti-malarial drug within 2 h of presentation; (3) no missing supplies and two instances of lack of blood in the hospital compared to 50 % lacking an essential drug or supply needed for resuscitation; (4) 92 vs 29 % highest parental satisfaction rating with medical care [30].

Safety was objectively and blindly assessed using standardized toxicity tables. The only clinical sign that differed between patients receiving iNO_80_ and placebo was peri-orbital oedema, which resolved without discontinuation of study gas. Increased interstitial fluid may be most evident in the loose areolar tissue around the eye in a recumbent patient, and may indicate transient alterations in vascular permeability [although there was no difference in Ang-2 levels), renal dysfunction with fluid retention (although was no difference in creatinine and rates of acute kidney injury (AKI)], or other reversible disturbances of capillary hydrostatic or oncotic pressures. As expected, levels of metHb rose in patients receiving iNO_80_. These levels could be controlled with downward titration of the study gas and required temporary discontinuation of iNO in only 5.7 % of patients. This dose-dependent adverse event provided evidence of biological activity of the administered therapy to alter the redox state of circulating haemoglobin, although an effect on the endothelium was not observed, based on Ang-2 measurements. AKI was identified as a toxicity of iNO in a meta-analysis pooling data from multiple randomized trials involving mostly adult patients, although this effect was not evident in individual studies [21]. The rate of AKI among patients in the present study was 8.0 % in the iNO compared to 3.3 % in the placebo group, which did not represent a statistically significant difference, although the present trial, like former randomized controlled trials of iNO, may have been underpowered to detect small differences in AKI rates. Co-treatment with diazepam for acute seizure management was statistically more frequent among patients receiving iNO. The significance of this finding is unclear since the proportion of patients with new onset or persistent seizures, frequency of neurologic sequelae, and time to recovery of consciousness was similar to placebo recipients.

Efficacy of iNO_80_ in a murine model of experimental cerebral malaria did not translate to efficacy in humans in this clinical trial; however, pre-clinical data from this model were critical for generating the hypothesis and designing the trial. In mice, unlike humans, iNO altered Ang-2 levels, decreased blood–brain barrier dysfunction, decreased brain accumulation of parasites, and improved survival [5]. Inter-species differences in iNO_80_ pharmacokinetics may account for lack of efficacy in the present trial. Rapid conversion of iNO to nitrate and other stable adducts may have reduced bioavailable NO at the endothelium. On the other hand, iNO has been shown to exert pharmacological effects beyond the pulmonary vasculature in other studies in humans [31]. Although no evidence of efficacy of iNO_80_ was observed, other doses, routes of administration or NO donor molecules remain viable options for future investigation. Likewise, the Ang-Tie2 pathway remains a valid target for experimental interventions, and the pre-clinical murine model remains a useful tool to test novel therapy in vivo prior to clinical trials.

## Conclusions

Attempts to find host-directed treatments for severe malaria over the past several decades have not yet yielded an effective adjunctive therapy. iNO_80_ did not affect mortality or alter the rate of change of Ang-2 in this study; however, targeting the endothelium to improve outcomes in severe malaria remains a viable strategy with broader implications for other life-threatening infections such as sepsis characterized by endothelial dysfunction. Alternative methods to increase NO bioavailability at the endothelial barrier (higher dose, donor molecules, route of delivery) deserve further investigation. Given the global burden of childhood malaria and the relatively high rate of morbidity and mortality despite treatment with anti-malarials, continued investigation of adjunctive therapy is warranted.

## Additional file

10.1186/s12936-015-0946-2 Sensitivity analyses and quality measures for clinical trial “Inhaled nitric oxide as adjunctive therapy for severe malaria”. Description: Sensitivity analyses on the primary and secondary (mortality) endpoints are provided. Measures of the quality of blinding and quality of clinical care in the trial are described.

## Abbreviations

AKI: acute kidney injury
Ang-2: angiopoietin-2
DSMB: data and safety monitoring board
ELISA: enzyme-linked immunosorbent assay
HRP2: histidine rich protein 2
HR: hazard ratio
iNO: inhaled nitric oxide
IQR: inter-quartile range
LME: linear mixed-effects
metHb: methaemoglobin
NO: nitric oxide
PE: parasitized erythrocyte
pLDH: parasite lactate dehydrogenase
WHO: World Health Organization
WPB: Weibel-Palade body
