# Supplementary material for: Inhaled nitric oxide as adjunctive therapy for severe malaria: a randomized controlled trial
Source: Malar J. 2015 Oct 29;14:421. doi: 10.1186/s12936-015-0946-2 (PMC4625637; doi:10.1186/s12936-015-0946-2)
Supplement: Supplementary file 1 — 10.1186/s12936-015-0946-2 Sensitivity analyses and quality measures for clinical trial “Inhaled nitric oxide as adjunctive therapy for severe malaria”. Description: Sensitivity analyses on the primary and secondary (mortality) endpoints are provided. Measures of the quality of blinding and quality of clinical care in the trial are described. [file 12936_2015_946_MOESM1_ESM.docx]

**Additional File 1**

**Sensitivity analyses and quality measures for clinical trial “Inhaled nitric oxide as adjunctive therapy for severe malaria”**

1. **Sensitivity analysis on primary endpoint (longitudinal Ang-2)**

In order to account for all randomized patients in the analysis (“intention-to-treat” analysis), it was necessary to adjust for missing Ang-2 measurements. There were a total of 48 missing data points (23/352=6.5% iNO and 25/368=6.8% placebo) among 18 patients (9/88=10.2% iNO and 9/92=9.8% placebo). Most data were missing due to mortality (17 data points among 6 patients in iNO group and 21 data points among 8 patients in placebo group). A smaller number of data were missing due to patient withdrawal from the study (4 data points among 1 patient in iNO group and 4 data points among 1 patient in placebo group), and missing samples (2 data points among 2 patients in iNO group).

Rigorous attempts to limit missing data at the trial design and conduct stage resulted in >90% of primary outcome data being available for analysis.^1^ Although it is unlikely that the small number of residual missing data would substantively affect conclusions of the analysis, we sought to adjust for missing data at the analysis stage in a sensitivity analysis, in order to demonstrate that our conclusions were robust to a variety of adjustments for the missing data.

Rational assumptions guided our handling of missing data, according to the mechanism by which the data was missing.^1^ We used linear interpolation to impute missing values for Ang-2 due to missing samples (data missing at random with flanking values for each of the 2 missing data points). We imputed missing values for patients who withdrew from the study using the pooled mean Ang-2 levels values from all remaining study patients. This assumption is conservative, since it would tend to bias toward the null hypothesis. For missing data due to mortality, we used several approaches: restrict analysis to survivors; arbitrarily carry forward the last available value for Ang-2; and arbitrarily assign increasing values of Ang-2 over time.

Under these various assumptions, we computed the linear rate of change of serum Ang-2 for each patient, with a comparison between treatment groups (table A1). The first and most straightforward analysis scenario used all available data, but may be subject to bias given that most data are not missing at random, but due to death. The second scenario (imputation of missing data for lost samples and withdrawals) resulted in no meaningful change to the estimates of rate of change of Ang-2. The third scenario (analysis restricted to survivors) is reasonable given that Ang-2 is not a meaningful outcome in patients who have died; furthermore, restricting the Ang-2 analysis to survivors is justified given that there was no difference in survival between iNO and placebo arms. The fourth scenario arbitrarily assigned the last available value of Ang-2 to patients after their death. This assumption is not easily justified, but results in minimal changes to the output of the analysis. Under the fifth scenario, arbitrarily rising Ang-2 levels were assigned to patients after death, at a rate of +9.7ng/mL/day, as observed in fatal cases in a previous study.^2^ Under none of these 5 scenarios was there a statistically significant difference in the linear rate of change of Ang-2 between iNO and placebo groups. Our conclusion that Ang-2 levels were not affected by the administration of iNO appears to be robust under multiple strategies to deal with (non-randomly) missing data.

**Table A1. Sensitivity analysis: effect of different approaches to missing data on estimate of linear rate of change of Ang-2.**

|  |  | **iNO** |  |  | **Placebo** |  | **p-value** |
| --- | --- | --- | --- | --- | --- | --- | --- |
|  | **observations**  **n (%)** | **patients**  **n (%)** | **linear change [ng/mL/day]**  **median (IQR)** | **Observations**  **n (%)** | **patients**  **n (%)** | **linear change [ng/mL/day]**  **median (IQR)** |  |
| **All available data** | 329 (93) | 82 (93) | -2.1  (-3.1 to -1.2) | 343 (93) | 86 (93) | -1.9  (-3.6 to -0.57) | 0.76 |
| **Imputed values for withdrawals and lost samples** | 335 (95) | 83 (94) | -2.2  (-3.0 to -1.2) | 347 (94) | 87 (96) | -1.9  (-3.6 to -0.59) | 0.77 |
| **Restricted to survivors** | 328 (93) | 82 (93) | -2.2  (-3.1 to -1.3) | 336 (91) | 84 (91) | -1.9  (-3.5 to -0.60) | 0.62 |
| **Mortality adjustment: last value carried forward** | 352 (100) | 88 (100) | -2.0  (-2.9 to -0.84) | 368 (100) | 92 (100) | -1.5  (-3.3 to -0.46) | 0.61 |
| **Mortality adjustment: increasing Ang-2** | 352 (100) | 88 (100) | -2.0  (-2.9 to -0.84) | 368 (100) | 92 (100) | -1.5  (-3.3 to -0.40) | 0.52 |

**2.** **Sensitivity analysis on secondary outcome (mortality)**

Mortality data (secondary outcome) was missing due to withdrawal from the study for 1 patient from the iNO group and 1 patient from the control group. The difference in mortality was not statistically significantly different between groups when all available data were analysed. This conclusion was robust to all possible unknown mortality outcomes in patients who withdrew from the study (p>0.2 for all possibilities).

**3.** **Quality of masking**

Quality of masking was assessed using a published “blindness index.”^3^ Masked respondents (parents/guardians of pediatric participants, study nurses, and study clinicians) were asked to guess the treatment assignment at hospital discharge for each patient, and answers were compared to the actual treatment assignment. The index varies between 0 (perfect guessing) and 0.5 (random guessing), since we did not allow for “don’t know” answers.^3^

The blindness index was 0.57 (95% CI 0.51-0.64; p=0.99), 0.42 (95% CI 0.34-0.49; p=0.015), and 0.49 (95% CI 0.42-0.57; p=0.44) for parents/guardians, nurses, and clinicians, respectively. This suggests that nurses, but not parents/guardians or clinicians, correctly guessed treatment allocation more frequently than would be expected by chance alone. Qualitative feedback from the nurses indicated that monitor alarms and titration of NO flow by the un-masked team were the principal cause of un-masking. No evidence of un-masking was observed for clinicians, who made all decisions with respect to co-treatments, and assessed outcomes, including timing of discharge. Furthermore, Ang-2 levels were objectively quantified using laboratory assays, and statistical analysis was performed in an entirely masked manner. Therefore, it is unlikely that significant bias was introduced through the accidental un-masking of trial nurses. Furthermore, the direction of any possible bias is not intuitively obvious, and is unlikely to explain the lack of effect of iNO_80_ observed in our trial.

**4. Quality of clinical care**

Quality of clinical care provided during the trial was assessed by several parameters: time from presentation until seen by clinician, time to initiation of artesunate, missing essential supplies, and parent satisfaction with care provided. In this cohort of critically ill patients presenting to the emergency department, the median [IQR] time to be seen by a clinician was 30 [0-60] minutes for iNO group and 21 [0-60] minutes for placebo group (p=0.60). The median [IQR] time from presentation to first dose of artesunate was 65 [33-150] minutes for iNO group and 70 [30-140] minutes for placebo group (p=0.78). There were no cases of missing supplies for treatment (IV cannula, syringe, artesunate, IV giving set, blood transfusion set, 25% dextrose), but in two patients with severe anemia (both iNO group), there was no matched blood in the hospital, and the patient required transfer to a private health facility for transfusion.

Parent satisfaction with clinical care was assessed at the time of hospital discharge. Parents gave the highest possible ranking (“very good”) on a five-point satisfaction scale for care on the study ward in 71/76 (93%) iNO group and 73/79 (92%) placebo group (p=0.81). Satisfaction scores for the study ward were higher than for care received in the emergency department (p<0.001). Compared to the national reference hospital in Uganda, our trial provided superior medical care, according to the following indices: (1) 77% *vs* 23% were seen by a clinician within one hour of presentation; (2) 68% *vs* 12% received the first dose of anti-malarial drug within 2 hours of presentation; (3) no missing supplies and 2 instances of lack of blood in the hospital compared to 50% lacking an essential drug or supply needed for resuscitation. (4) 92% *vs* 29% highest parental satisfaction rating with medical care.^4^

**References**

1. Little RJ, D'Agostino R, Cohen ML, et al. The prevention and treatment of missing data in clinical trials. *N Engl J Med* 2012; **367**(14): 1355-60.

2. Yeo TW, Lampah DA, Gitawati R, et al. Angiopoietin-2 is associated with decreased endothelial nitric oxide and poor clinical outcome in severe falciparum malaria. *Proc Natl Acad Sci U S A* 2008; **105**(44): 17097-102.

3. James KE, Bloch DA, Lee KK, Kraemer HC, Fuller RK. An index for assessing blindness in a multi-centre clinical trial: disulfiram for alcohol cessation--a VA cooperative study. *Stat Med* 1996; **15**(13): 1421-34.

4. Idro R, Aloyo J. Manifestations, quality of emergency care and outcome of severe malaria in Mulago Hospital, Uganda. *Afr Health Sci* 2004; **4**(1): 50-7.
